# Machine Learning and Deep Learning in Cardiothoracic Imaging: A Scoping Review

**DOI:** 10.3390/diagnostics12102512

**Published:** 2022-10-17

**Authors:** Bardia Khosravi, Pouria Rouzrokh, Shahriar Faghani, Mana Moassefi, Sanaz Vahdati, Elham Mahmoudi, Hamid Chalian, Bradley J. Erickson

**Affiliations:** 1 Radiology Informatics Lab (RIL), Department of Radiology, Mayo Clinic, Rochester, MN 55905, USA; 2 Orthopedic Surgery Artificial Intelligence Laboratory (OSAIL), Department of Orthopedic Surgery, Mayo Clinic, Rochester, MN 55905, USA; 3 Department of Radiology, Cardiothoracic Imaging, University of Washington, Seattle, WA 98195, USA

**Keywords:** artificial intelligence, machine learning, deep learning, cardiothoracic imaging, scoping review, radiology

## Abstract

Machine-learning (ML) and deep-learning (DL) algorithms are part of a group of modeling algorithms that grasp the hidden patterns in data based on a training process, enabling them to extract complex information from the input data. In the past decade, these algorithms have been increasingly used for image processing, specifically in the medical domain. Cardiothoracic imaging is one of the early adopters of ML/DL research, and the COVID-19 pandemic resulted in more research focus on the feasibility and applications of ML/DL in cardiothoracic imaging. In this scoping review, we systematically searched available peer-reviewed medical literature on cardiothoracic imaging and quantitatively extracted key data elements in order to get a big picture of how ML/DL have been used in the rapidly evolving cardiothoracic imaging field. During this report, we provide insights on different applications of ML/DL and some nuances pertaining to this specific field of research. Finally, we provide general suggestions on how researchers can make their research more than just a proof-of-concept and move toward clinical adoption.

## 1. Introduction

Artificial intelligence (AI) is a broad term used to define systems that perform tasks that typically require human intelligence [1]. Early efforts to create such systems in medicine focused on using known relationships, casualties, and decision logic to develop an “intelligent” algorithm, much like diagnostic flowcharts but executed by a computer. In the 21st century, with the widespread adoption of electronic health records (EHR), “big data” came into existence [2]. Big data refers to massive multidimensional collections of information about each individual, for example, their demographic information, lab test results, medication history, and imaging studies, just to name a few. Using traditional statistical methods to interpret this data is not optimal, as these methods cannot capture the complexities of multidimensional data [3]. Therefore, researchers turned to machine-learning (ML) algorithms to harness this information.

ML is a subfield of AI that encompasses algorithms that “learn” the relationships between data elements by seeing many examples [4]. This makes ML algorithms data-driven, meaning that the input data’s quality (and quantity) determines their performance. Deep learning (DL) is a group of ML algorithms that extract relationships in the data through multilayer neural networks, resembling a human cognition system [5]. Due to their hierarchical architecture, DL algorithms have the capacity to abstract complex information from images. They have proven their potential by outperforming humans in several natural-image tasks, such as handwritten image classification and face detection [6,7,8].

Medical imaging is the perfect place to harness the power of these ML and DL algorithms. As an example, just in 2016, there were more than 75 million computed tomography (CT) examinations performed in the United States [9]. Considering this number and the vast information in each study, the potential to use ML algorithms to extract information to help diagnose different diseases is immense. Additionally, ML and DL can be used to extract parameters from the image to predict the prognosis or survival of a patient or optimize clinical workflows [10]. There are other potential applications for DL in image reconstruction, which are listed in the applications section in the results of this report.

Developing these algorithms requires some programming background, but, in recent years, there have been some no-code solutions, called *AutoML*. These allow researchers to focus on the input data, and the rest, including model development and fine-tuning, is taken care of by these AutoML softwares. These produce results comparable to manually developed models and have lowered the bar to use ML/DL algorithms by clinical researchers [11].

Several studies have explored radiologists’ attitudes toward the progression of AI in their field and their knowledge about it [12]. In a survey by Huisman et al. of more than 1000 participants, 69% reported some knowledge of AI, with 11% denoting their knowledge as advanced [13]. In contrast, Ooi et al. reported that 65% of their participants evaluated their knowledge of AI as novice at best [14]. Despite these gaps in knowledge, radiologists have a consensus that AI will dramatically impact their field in ten years or even sooner [15,16,17]. Additionally, in several reports, radiologists have expressed that they do not believe AI will replace them in the foreseeable future [14,18,19]. The model usually proposed is a cooperation of AI and radiologists, with AI facilitating and increasing the efficiency of radiologists and improving the diagnostic and prognostic workflows [20]. A radiologist might not need to know the technical details of ML/DL algorithms; however, knowing some general concepts would help one prepare for an even-more technology-rich practice.

Cardiothoracic imaging is one of the early adopters of ML/DL algorithms. This is mainly attributed to the public release of large datasets early in the days that DL was gaining attraction in the medical field [21]. These datasets provided a valuable resource for researchers and developers outside the medical field to test and benchmark their algorithms. Additionally, with the COVID-19 pandemic, an arsenal of researchers have tried to apply computer-aided diagnostic tools to help with the early diagnosis of the disease and predict the prognosis of patients [22]. With the variety of modalities used in cardiothoracic imaging, there is a potential to help augment radiologists’ abilities in their routine clinical practice.

In this report, we review papers on cardiothoracic image analysis using AI and try to paint a big picture of advances in this field. For the presented results, we highlight some nuances of applying AI to medical problems, specifically cardiothoracic imaging. Finally, we summarize the findings by providing some useful tips for radiologists interested in performing applied AI research.

## 2. Materials and Methods

In this review, we addressed *how ML/DL are used in cardiothoracic imaging*. Inclusion criteria included (a) peer-reviewed articles on (b) cardiothoracic imaging, with (c) English full-text, that (d) have used ML or DL algorithms in their study. We excluded articles (a) focusing on non-imaging studies, specifically pathology images, and (b) all non-original research papers, including review articles or systematic analyses. We followed the Preferred Reporting Items for Systematic Reviews and Meta-Analysis (PRISMA) extension for scoping reviews [23].

To identify relevant studies, a systematic search was done in the MedLine database. The search included three parts, one to include organs that are located in the thoracic area, another to target the ML/DL component of their study, and, finally, a part to search for relevant modalities. Supplementary Table S1 shows the terms used in this phase. Additionally, due to the effect of COVID-19 on the number of publications both in the cardiothoracic imaging field in general and also AI research, we included the term “COVID-19” to encompass the latest advancements in this field [24]. The search was confined to the period 1 January 2012 to 31 May 2022 to be inclusive of the most recent advances in the field. The beginning timepoint was selected as the first scalable convolutional neural network (i.e., a subclass of DL models that work on image analysis) introduced back in 2012 and significantly improved the state-of-the-art results in natural-image classification [25]. The search was initially carried out on 15 July 2022 and later updated on 27 August 2022.

For each study, 11 data items were extracted to answer our research question and give us a broad understanding of this field. Extracted data items included study type, sample size, ML/DL task, use of external validation, and ability to explain, as detailed in Table 1. Initially, a set of 50 studies were selected, and their characteristics were extracted by the authors and discussed as a group to level their understanding of the required fields. After reaching a consensus on this subset, the authors independently extracted data from the remaining articles. During this process, any ambiguities were checked with others in a private online forum. Due to a large number of remaining studies, we were not able to assess the bias of the individual articles. Data analysis and plotting were done using Python (v3.9; Python Software Foundation, USA) and the Plotly library (v5.10.0; Plotly Inc., USA) [26].

In the synthesis section, we did a thematic analysis of the extracted information. For the presented results, we first give some general information on that theme and why it is important and then report our findings.

## 3. Results and Synthesis

**Figure 1:**
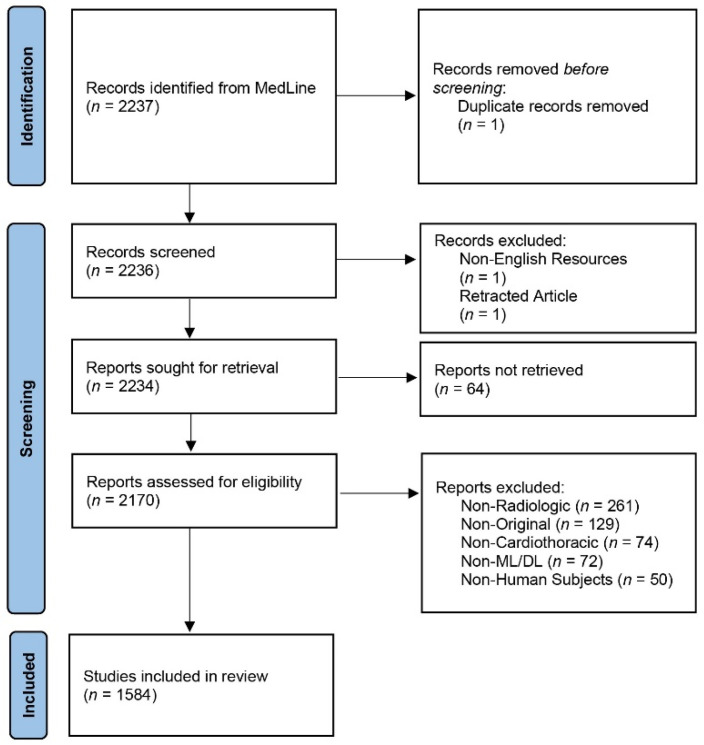
PRISMA inclusion flow chart.

**Figure 2:**
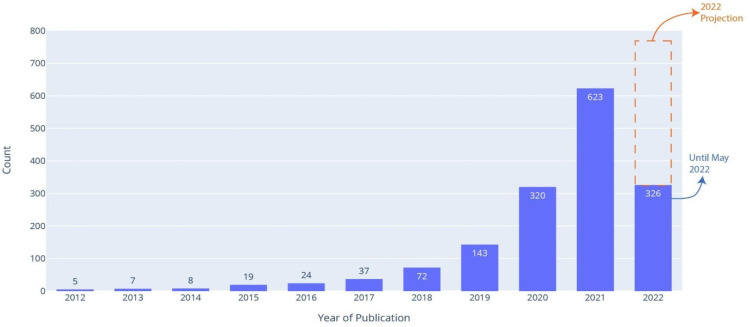
Yearly distribution of included studies.

**Figure 3:**
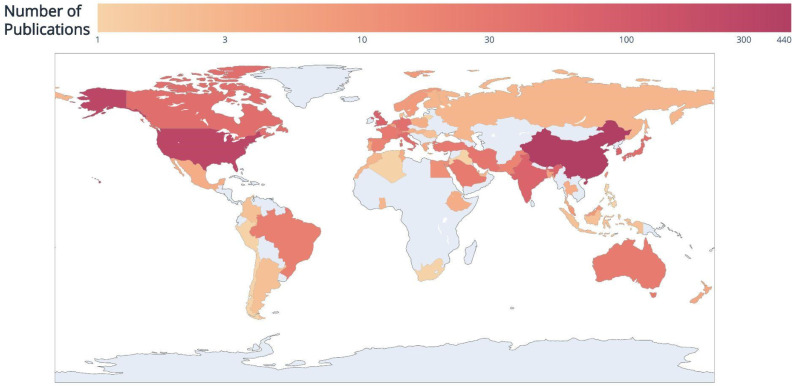
Geographical distribution of included studies. The scale is log-based to better visualize the variability between different countries.

The systematic search yielded 2237 manuscripts. After careful examination, 652 were excluded yielding a final number of 1584 studies that met our eligibility criteria, detailed in Figure 1. From 2012 to 2021, the number of published studies increased year by year, with large increases in 2020 and 2021, as seen in Figure 2. This can be attributed to the COVID-19 pandemic and the resulting influx of publications on this topic [27]. Moreover, based on the first five months, the forecasted number of publications in 2022 was around 780, which is less than the yearly doubling trend seen since 2017, confirming the decrease of the “COVID publication fever”. As shown in Figure 3, China, the United States, and South Korea have the highest number of publications in this field, with 444, 290, and 82 manuscripts, respectively.

### 3.1. Clinical Application

**Figure 4:**
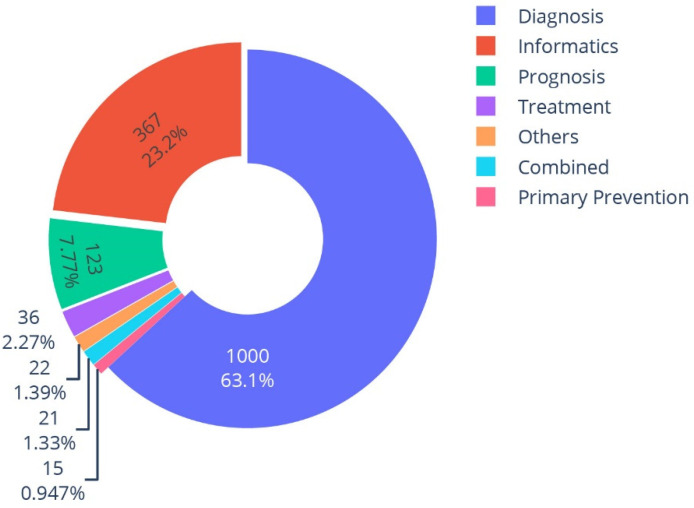
Distribution of clinical applications of the included studies.

Among the included studies, 1000 focused on disease diagnosis (63%) and 123 (8%) studies targeted patient prognosis, as seen in Figure 4. Three hundred sixty-seven studies (23%) worked on organ segmentation or image quality improvement and were categorized as informatics-related. This segmentation can be further utilized in downstream tasks; for example, Qi et al. used a deep-learning algorithm to segment lung nodules and used the masks for longitudinal surveillance of lung-cancer patients [28]. It is noteworthy to mention that if an informatics task, like segmentation, was utilized for a clinical purpose, the study was categorized based on the clinical application.

### 3.2. Organ and Pathology

Most of the studies focused on the lung as their primary organ of interest, comprising 1025/1584 (65%), and 514/1584 (32%) of studies worked on the cardiovascular system. Ten studies worked on multiple-organ systems. The majority of studies worked on COVID-19 and lung-nodule detection and classification, as seen in Table 2.

COVID-19 was the most commonly studied pathology among the reviewed studies. As the pandemic started, pulmonary CT images and chest radiographs were regarded as first-line screening and diagnostic tools [29]. Although soon reverse transcription polymerase chain reaction (RT-PCR) replaced imaging studies as the gold standard of diagnosis, the AI community was very eager to test their algorithms to see how far they could push the limits of COVID-19 diagnosis based on imaging features [30]. Additionally, many studies used ML/DL to prognosticate patients with COVID-19 and predict severe outcomes, like ICU admission or death [31]. Publicly available datasets and coding challenges fueled this enthusiasm by creating a way to benchmark algorithm performance [32,33,34].

Lung-nodule detection has gained attention since one of the first large-scale medical datasets that was publicly released was the LUng Nodule Analysis challenge (LUNA) dataset, which contains 888 lung CT series with the exact location of each nodule [21]. The other publicly available dataset is the Lung Image Database Consortium image collection (LIDC-IDRI), which is comprised of 1018 lung CT examinations with each nodule being segmented by cardiothoracic radiologists and a subset (157 patients) labeled as malignant or benign based on pathology reports [35]. These public datasets have paved the way for non-medical researchers to work on these challenging tasks by providing annotated high-quality data, which can be the most important hurdle in a machine-learning project.

Among studies focusing on the heart as their primary organ of interest, the majority were classified in the realm of informatics. For example, Carbaja-Degante et al. focused on ventricle segmentation on cardiac CT and MRI [36]. Additionally, many studies have tried to calculate the calcium scoring of coronary arteries based on cardiac CTs [37,38].

### 3.3. Imaging Modality

**Figure 5:**
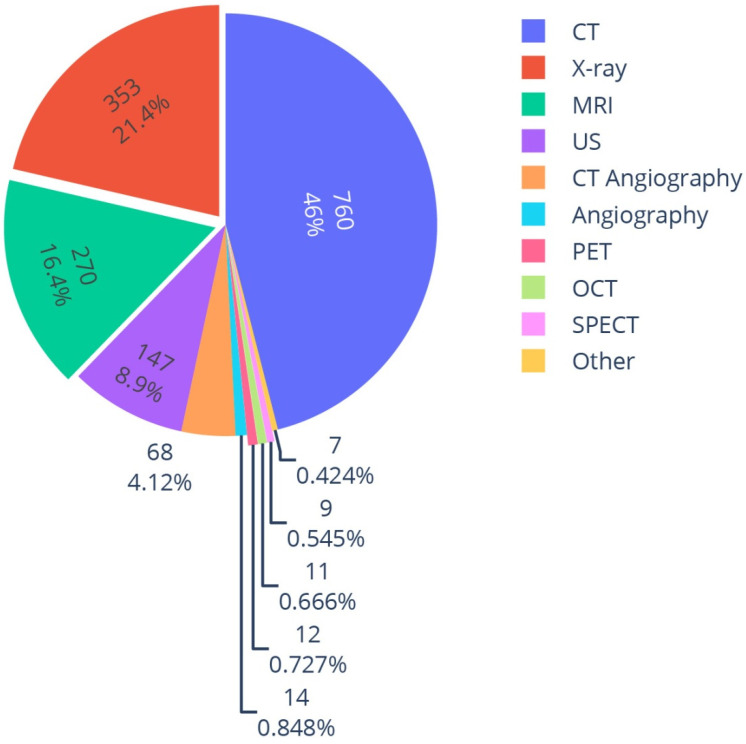
Distribution of target modalities of the included studies.

A majority of the studies used CTs for their image analysis, comprising 760/1651 (46%). As 67 studies used two different modalities, the denominator of the fractions in this section is 1651, i.e., the total number of modalities used, rather than 1584 (the number of included studies), as seen in Figure 5. This might be attributed to the fact that there are many publicly available CT imaging datasets, as discussed previously. Some studies feed slices of the 3D volume to a 2D model, essentially increasing their training data, with the cost of losing volumetric information. To preserve spatial information, one can use a 3D model. As one might think, using a full 3D volume to train a DL model needs a high-performing computational infrastructure. One way to overcome the computational challenge is an approach called *2.5D training*. As an example, Kim et al. first segmented lung nodules in CT images [39]. Then, using 3D regions of interest for the segmented nodules, they obtained nine different 2D images in the axial, coronal, and sagittal planes with two additional oblique cross sections (45° and −45°) per plane. Finally, they combined them into one image that is not truly 3D or 2D; hence, it is called 2.5D. They showed that for the specific problem of differentiating adenocarcinomas, the 2.5D approach outperformed the 3D approach and was comparable to radiologists’ performance with much lower computational costs compared to 3D.

X-ray radiographs are the second most common modality used in the included ML/DL studies. This can be attributed to the fact that radiographs are very common in both developed and developing countries, and many diseases can be diagnosed based on this 2D image. Additionally, there are several large-scale public datasets on chest X-rays, namely the CheXpert dataset and the NIH chest X-ray dataset [40,41]. When combined, these two datasets provide more than 300,000 images, each labeled for the presence of several conditions, including pleural effusion, pneumonia, pneumothorax, and nodules.

### 3.4. Machine-Learning-Algorithm Type and Sample Size

**Figure 6:**
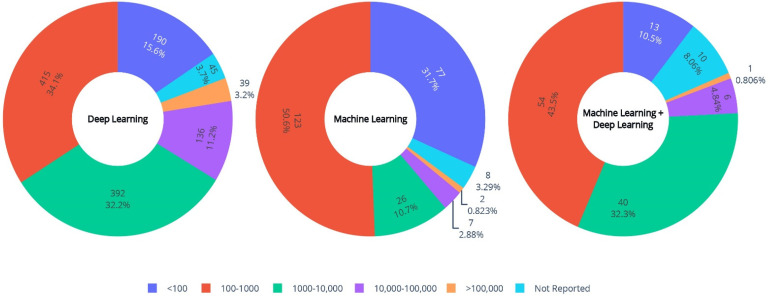
Distribution of different machine-learning methodologies and sample size of the included studies.

A total of 1217/1584 (77%) studies used deep learning, and only 242/1584 studies (15%) used conventional machine learning for cardiothoracic image analysis; the other 125 (8%) used a combination of conventional machine learning and deep learning, as seen in Figure 6. As expected, studies utilizing DL had a higher number of imaging samples, as the final performance of these models highly depends on the quantity and quality of the training data. This should be noted because many studies only mentioned the number of included patients, which might not always be equal to the number of examinations used for training and might be misleading to their readership.

Conventional machine-learning algorithms are known to work well with tabular data. One popular approach to use these models for image analysis is by utilizing radiomics features [42]. Radiomics is a method for quantitatively describing medical images; in contrast to radiologists who might report a pulmonary nodule as “...a 3 mm perifissural nodular opacity within the lingula…”, radiomics describes the nodule with numerical values contributing to the overall shape, texture, sphericity, contrast with surrounding tissue, etc. [43]. To perform radiomics-feature extraction, one has to segment the region of interest, either manually or by deep-learning algorithms, and run predefined algorithms on that region [44]. The output is a high-dimensional table filled with numerical values, which is the input to a conventional ML model.

One approach that enables using DL algorithms to perform well in limited data settings is a technique called *transfer learning*, in which the parameters of an already-trained DL model are used in a downstream task [45]. There are plenty of publicly available models that are pretrained on natural or medical images that can be used for feature extraction for downstream tasks [46,47]. Similarly, Chen et al. used such a pretrained DL model, applied the model to endobronchial ultrasound images (without retraining), extracted features, and used a machine-learning algorithm to predict malignancy of the pulmonary nodules [48]. This technique enabled them to reach an area under the receiving operating curve (AUROC) of 0.87 on only 164 patients.

### 3.5. Machine Learning Tasks

ML and DL tasks can be divided into five major categories.

#### 3.5.1. Classification

The goal of classification is to assign a single label to the whole image, for example, whether a chest X-ray shows signs of pneumonia or not. These tasks can be binary (i.e., yes/no labels), multiclass (selecting one option from more than two choices that are mutually exclusive, e.g., viral pneumonia/bacterial pneumonia/COVID), or multi-label (where there is potential for the co-occurrence of conditions, e.g., having both cardiomegaly and pleural effusion) [49,50,51,52]. Of the included studies, 869 used a classification model in a part of their pipelines. Of these, 190 studies combined classification with other types of ML tasks, for example, first segmenting a lung nodule and then classifying the isolated nodule as benign or malignant [53].

#### 3.5.2. Regression

Regression tasks generate a continuous numerical value from the input data. A common example is predicting the survival of patients with lung cancer; it should be noted that by using noncontinuous time periods in survival analysis, the task becomes a multiclass classification [54]. Eighty-eight studies used regression in their analysis pipelines. Other use cases of regression tasks in cardiothoracic imaging include measurement of aortic calcium score, ventricle volume, and pulmonary lesion volume [55,56,57,58].

#### 3.5.3. Object Detection

Sixty-six studies did object detection, which is localizing an object of interest in an image using key points or bounding boxes. For example, Rafael-Palou et al. used a deep-learning network to localize lung nodules and then used this model for automated follow-up of patients with pulmonary nodules [59]. In another example, Pezzano et al. trained an object-detection deep-learning-based model to determine the location of COVID-19 opacities, which can further be used for calculating severity scores for patients [60].

#### 3.5.4. Semantic Segmentation

Semantic segmentation involves exact delineation of the organ of interest by an ML model, and was performed in 529 studies. To highlight the power of deep-learning algorithms in segmentation tasks, Nardelli et al. used a DL model to separate arteries and veins in pulmonary vasculature in CT scans [61]. Other instances of segmentation in cardiothoracic imaging involves delineating ventricular myocardium or valve leaflets that can be used to calculate structural and flow-related parameters [62,63,64,65]. Segmentation tasks require experts to annotate the organ of interest, which makes preparing the training data both time-consuming and expensive. Some techniques can help with reducing the required training data without hurting the model performance, like Few-Shot Learning. For example, Wang et al. used this technique to achieve a very accurate heart segmentation model with only four annotated CT angiograms [66].

#### 3.5.5. Generative Tasks

In this broad category of ML tasks, the model is used to create images similar to, but not exactly the same as, the original input data. For example, these models have been used to increase image resolution or create a dual-energy X-ray from traditional X-rays [67,68]. Overall, 125 studies used generative approaches in their methodology design. An intuitive use case of generative models is artificially increasing low prevalence classes in the dataset by creating synthetic images with those characteristics. Astaraki et al. used this technique to create synthetic images of pulmonary nodules based on a limited dataset in order to train a segmentation model [69]. They showed that the trained segmentation model performs well on real patient images, highlighting the power of DL-based image generation. Another popular application of generative models in cardiothoracic imaging is creating standard-dose scans from low-dose sequences [70,71].

### 3.6. External Validation

The most important issue that hinders ML/DL algorithm adoption in healthcare is their lack of generalizability, meaning that an algorithm can perform well on a particular set of data (similar to the one it was trained on) while having suboptimal performance on external data [72]. This potential drawback necessitates rigorous validation of these algorithms on external data, called external validation. Without this process, one cannot trust if the reported performance of the model can happen in other real clinical cases [73,74]. Of the reviewed studies, 245 (15%) utilized external validation to test the generalizability of their models. Source that can be used for external validation are the publicly available datasets; in which case, the algorithm is trained on the institutional data, and then its performance is evaluated on the public set, allowing fair comparison between the different algorithms [75,76,77].

### 3.7. Interpretability Maps

DL algorithms are prone to biases that are caused by the model taking “shortcuts” rather than focusing on important features. In an interesting study, Rueckel et al. evaluated this phenomenon for two publicly available pneumothorax-detection algorithms [78]. They have shown that these algorithms actually detect the inserted chest tube on the X-rays as a surrogate marker for pneumothorax, causing substantial performance differences in patients with and without chest tubes. This bias can especially be present in many DL-based classification/regression algorithms, as they give a label to the whole image, but the basis for its decisions is often difficult to obtain. Therefore, these DL models are sometimes referred to as black boxes.

There are some ways that researchers can gain insight into how DL networks reach a particular decision, which is called interpretability maps [79]. Though these maps can help with the adoption of DL models in clinical settings, they are not perfect themselves and might cause biases [80]. Teng et al. provide a decent overview of how these tools work and how they can be used in medical image analysis [81]. Further, they only provide location information and not the reason that location was selected. Of the 694 studies that used only classification or regression in their image-analysis pipelines, 184 presented some visual explanations of how the model reached its decision.

## 4. Discussion

Artificial intelligence, and specifically deep learning, is a rapidly evolving field of computer science. As these algorithms can help with task automation, they have also gained attraction in the medical domain. In this scoping review, we aimed to provide a holistic view of ML and DL applications in cardiothoracic imaging, while summarizing some important nuances of this field. As evidenced by our findings, there is a growing number of publications in cardiothoracic imaging, and the COVID-19 pandemic sparked a big jump in the total number of studies. Researchers have used ML/DL to solve many clinical problems, with a higher focus on disease diagnosis. Having said this, some factors hinder the adoption of these algorithms in clinical practice. Based on this review, here are some future directions that ML researchers can pursue in the field of cardiothoracic imaging.

### 4.1. Generalizability Testing

Although a limited number of the reviewed studies externally validated their algorithm, most of the included studies did not test the generalizability of their models on external data. This is a crucial step toward showing a proof-of-concept study that can be useful in real clinical scenarios. It is of utmost importance to use data from different sources as the training data, not just setting aside a portion of training data for testing purposes, like many of the reviewed studies did. This causes inherent biases in the training data being present in the test set, causing a misleadingly high performance [82]. One solution would be using publicly available datasets as a community-assigned benchmark. Although this means refraining from using these datasets during training, it ensures comparable results across different studies, but it would also demand these public datasets be representative of the populations where the algorithm is deployed.

### 4.2. Applied Research

As many radiologists believe, AI is here to help them with their day-to-day clinical workflows, rather than replacing them. However, not many studies tested these algorithms side-by-side to a radiologist, and those who did compared them against each other, rather than in a cooperative-reading setting [83,84]. For AI to be integrated into the clinical workflow, extensive studies are needed to quantify the AI’s help and to show its efficiency and efficacy in prognostic and diagnostic workflows. As an example, it is controversial whether a radiologist should read a study and then see the AI’s prediction, or the prediction results should be shown to the radiologist in the first place [85]. These questions should be carefully investigated by researchers in order to facilitate AI adoption.

Our study is subject to several limitations that need to be addressed in future efforts. First, as we wanted to give a general overview of the field, we only used organ keywords from Medical Subject Headings (MeSH terms) in our search query. Such a strategy might have caused us to miss some studies, but we believe our search is still representative of the whole literature. Second, we only searched MedLine as the main database of medical literature; however, many AI researchers might release non-peer-reviewed versions of their work on preprint servers, like arXiv or medRxiv. Finally, because there was a need to include numerous studies to reach the goal of this scoping review, we could not assess the biases of the included papers, as it would make the task intractable. We strongly encourage future studies focusing on a specialized subfield of cardiothoracic imaging to identify hidden biases in ML/DL research in this field.

## 5. Conclusions

Overall, ML and DL have gained attraction in cardiothoracic imaging research, especially after the COVID pandemic. Researchers have utilized these techniques to analyze a variety of modalities in order to diagnose diseases, monitor treatment, enhance imaging quality, and predict patient prognosis. As a rule of thumb, DL models require more training data compared to other conventional ML algorithms; however, there are some techniques like transfer learning or Few-Shot Learning that can help alleviate this problem. There are also ML designs, like radiomics studies that extract handcrafted features from medical images or creating tabular data ready to be fed to an ML model, which can be done in data-constraint settings. With all these advances, there are some less-explored areas, like testing generalizability, quantifying model uncertainty, and evaluating the effect of AI in radiologists’ workflows and decision-making processes that need to be further investigated by ML researchers. While there have been tremendous advances in AI technology in the recent past, it is critical that AI researchers recognize the potential pitfalls of both the AI technology itself and the challenges of integrating these tools into clinical practice.

## Figures and Tables

**Table 1:** Extracted characteristics.

Characteristic	Description
Year	The year it was published based on MedLine database
Number of Subjects	Binned into categories of <100, 100–1000, 1000–10,000, 10,000–100,000, and >100,000
Country of the Authors	The affiliation country of the corresponding author
Clinical Application Type	Either diagnosis, treatment, prognosis, informatics, combined, or other
Study Modality	Modality or modalities that were used in the study
Studied Organ	The organ that was studied
Studied Disease	If there was a specific pathology that the study targeted
ML Methodology Category	Either conventional machine learning, deep learning, or a combination of both
ML Task Type	Classification, regression, segmentation, object detection, image generation, or other (multi-option)
Use of External Validation	If they used external data to validate their pipeline
Use of Explainable Methods	If they used any explainable method (only for deep-learning-based studies with classification or survival analysis tasks)

**Table 2:** Distribution of organs of interest and investigated pathologies. Note that several studies worked on multiple pathologies from different organs.

Organ	Pathology	Count
Lung	COVID-19	551
	Malignancy	265
	Interstitial Lung Diseases	93
	Infection (non-COVID-19)	89
	Obstructive Lung Diseases	82
	Pneumothorax	61
	Pulmonary Edema	58
	Pleural Effusion	56
	Atelectasis	53
	Tuberculosis	10
	Respiratory Distress Syndrome	6
	Cystic Fibrosis	5
	No Specific Pathology	87
Heart	Coronary Artery Disease	114
	Cardiomegaly	90
	Valvular Disorders	28
	Heart Failure	26
	Cardiomyopathy and Myocardial Disease	21
	Arrhythmia	15
	Congenital Heart Diseases	6
	Fat Analysis	5
	Pericarditis	1
	No Specific Pathology	195
Vascular System	Aortic Aneurysm and Dissection	6
	Pulmonary Hypertension	3
	Coarctation of the Aorta	1
	No Specific Pathology	8
Chest Wall	No Specific Pathology	11
Lymphatic System	Malignancy	4
	No Specific Pathology	1
Thymus	Malignancy	2

## Data Availability

Data can be shared upon reasonable request from the corresponding author.

## Supplementary Materials

The following supporting information can be downloaded at https://www.mdpi.com/article/10.3390/diagnostics12102512/s1, Table S1: Different parts of the search term that were used for retrieving relevant studies.
