# Supplementary material for: Machine Learning and Deep Learning in Cardiothoracic Imaging: A Scoping Review
Source: Diagnostics (Basel). 2022 Oct 17;12(10):2512. doi: 10.3390/diagnostics12102512 (PMC9600598; doi:10.3390/diagnostics12102512)
Supplement: Supplementary file 1 [file diagnostics-12-02512-s001.zip › diagnostics-1949028-supplementary.pdf]

Table S1. Different parts of the search term that were used for retrieving relevant studies.

**Cardiovascular Keywords**
(“heart”[MeSH] OR “Aorta”[Mesh] OR "Coronary Vessels"[Mesh] OR “lung”[MeSH] OR "Thorax"[Mesh] OR "Thymus Gland"[Mesh] OR “COVID-19”[MeSH])
**Artificial Intelligence Keywords**
(((“Machine Learning”[MeSH] OR “Neural Networks, Computer”[MeSH] OR “naive bayes”[tiab] OR “bayesian learning”[tiab] OR “neural network*”[tiab] OR “random forest”[tiab] OR “deep learning”[tiab] OR “machine prediction”[tiab] OR “machine intelligence”[tiab] OR “generative adversarial networks”[tiab] OR “Hierarchical Learning”[tiab] OR “computer vision”[tiab] OR “computational intelligence”[tiab] OR “computational learning”[tiab] OR “computer reasoning”[tiab] OR “machine learning”[tiab] OR “reinforcement learning”[tiab] OR “convolutional network*”[tiab] OR “artificial intelligence”[tiab] OR “Self Organizing MAP”[tiab] OR “Self-Organizing MAP”[tiab] OR “AutoEncoder”[tiab] OR “CNN”[tiab] OR “GAN”[tiab] OR “GANN”[tiab])) OR ((“convolute”[All Fields] OR “convoluted”[All Fields] OR “convolutes”[All Fields] OR “convoluting”[All Fields] OR “convolution”[All Fields] OR “convolutional”[All Fields] OR “convolutions”[All Fields] OR “convolutive”[All Fields] OR “transformer”[All Fields]) AND (“neural networks, computer”[MeSH Terms] OR (“neural”[All Fields] AND “networks”[All Fields] AND “computer”[All Fields]) OR “computer neural networks”[All Fields] OR (“neural”[All Fields] AND “network”[All Fields]) OR “neural network”[All Fields])))
**Imaging Keywords**
((“Diagnostic Imaging”[MeSH] OR “Image Processing, Computer-Assisted”[MeSH] OR “Imaging” OR “Radiograph*” OR “x?ray” OR “Tomograph*” OR “Magnetic Resonance” OR “MR?image*” OR “MRI” OR “MRA”[tiab] OR “CT?Scan*” OR “Ultrasonograph*” OR “Ultrasound*” OR “Echo*” OR "Echocardiography"[Mesh] OR “PET?Scan” OR “c-arm” OR “fluoroscop*” OR “cone?beam CT” OR “image-guided adaptive radiation therapy” OR “IGART”[tiab]))
